# Reliability and validity of the Children’s Fear Survey Schedule-Dental Subscale for Arabic-speaking children: a cross-sectional study

**DOI:** 10.1186/s12903-016-0205-0

**Published:** 2016-04-14

**Authors:** Azza A. El-Housseiny, Farah A. Alsadat, Najlaa M. Alamoudi, Douaa A. El Derwi, Najat M. Farsi, Moaz H. Attar, Basil M. Andijani

**Affiliations:** Pediatric Dentistry Department, Faculty of Dentistry, King Abdulaziz University, PO Box 80209, Jeddah, 21589 Saudi Arabia; Pediatric Dentistry Department, Faculty of Dentistry, Alexandria University, Alexandria, Egypt; Public Health and Community Medicine Department, Faculty of Medicine, Cairo University, Cairo, Egypt

**Keywords:** Child dental fear, CFSS-DS, Arabic version, Reliability and validity

## Abstract

**Background:**

Early recognition of dental fear is essential for the effective delivery of dental care. This study aimed to test the reliability and validity of the Arabic version of the Children’s Fear Survey Schedule-Dental Subscale (CFSS-DS).

**Methods:**

A school-based sample of 1546 children was randomly recruited. The Arabic version of the CFSS-DS was completed by children during class time. The scale was tested for internal consistency and test-retest reliability. To test criterion validity, children’s behavior was assessed using the Frankl scale during dental examination, and results were compared with children’s CFSS-DS scores. To test the scale’s construct validity, scores on “fear of going to the dentist soon” were correlated with CFSS-DS scores. Factor analysis was also used.

**Results:**

The Arabic version of the CFSS-DS showed high reliability regarding both test-retest reliability (intraclass correlation = 0.83, *p* < 0.001) and internal consistency (Cronbach’s α = 0.88). It showed good criterion validity: children with negative behavior had significantly higher fear scores (*t* = 13.67, *p* < 0.001). It also showed moderate construct validity (Spearman’s rho correlation, *r* = 0.53, *p* < 0.001). Factor analysis identified the following factors: “fear of invasive dental procedures,” “fear of less invasive dental procedures” and “fear of strangers.”

**Conclusion:**

The Arabic version of the CFSS-DS is a reliable and valid measure of dental fear in Arabic-speaking children. Pediatric dentists and researchers may use this validated version of the CFSS-DS to measure dental fear in Arabic-speaking children.

## Background

Dental fear is a key factor that may cause patients to avoid, delay, or even cancel dental appointments, leading to irregular attendance patterns [1]. Early recognition of children’s dental fear is essential to effective dental treatment [2, 3].

Dental fear in children may be measured using various methods, including behavioral ratings during dental visits, such as the Frankl behavioral rating scale, [4] physiological methods (e.g., pulse rate, muscle tension), and psychometric assessment (i.e., questionnaires completed either by a parent or by the children themselves) [5]. Psychometric assessment includes the Children’s Fear Survey Schedule-Dental Subscale (CFSS-DS), [6] which is the most widely used scale for children [5, 7]. The CFSS-DS rates children’s dental fear in 15 dentally related situations, such as “dentist,” “injections,” and “opening the mouth” [6]. Responses use a 5-point Likert scale; possible scores range from 15 to 75.

The CFSS-DS has been used in numerous countries and translated into Dutch, [8] Finnish, [9] Japanese, [10] Greek, [11] Chinese, [3] Hindi, [12] and Bosnian [13]. It is one of the most commonly used fear scale that has undergone reliability and validity testing in multiple languages [7]. It has high internal consistency and test-retest reliability, and satisfactory criterion and construct validity in English and many other languages [6, 9–11, 13, 14].

Recently, an Arabic version of the CFSS-DS has been developed and preliminarily validated in a clinically based sample [15, 16]. Since such samples are not representative and do not include children who avoid visiting dentists due to fear, further validation is required. A validated fear scale will permit the collection of normative data from Arabic-speaking children worldwide, and will enrich fear research. This study aimed to test the reliability and validity of the Arabic version of the CFSS-DS in primary school children.

## Methods

### Sample

In total, 198,405 children were enrolled in 624 primary schools (278 boys’ schools and 346 girls’ schools) in Jeddah. In order to determine the minimum satisfactory sample size, the prevalence of fear in the target population was hypothesized to be 20 % based on previous studies in different populations [8, 9]. Therefore, in the sample size calculation, the percentage frequency of outcome factors was set at 20 % with ± 2 % confidence limits. In addition, the confidence level was set at 95 %, the level of significance at 0.05, and power at 85 %. Sample size was calculated using the free web-based operating system OpenEpi, version 2 [17]. The estimated necessary sample size was 1525 children.

This was a multistage stratified random sample of children attending primary schools. Using the Jeddah city school list for 2013–2014, school selection was stratified by district (geographical location in Jeddah city), gender (boys or girls), and school type (public or private).

The determined sample of 1525 children was distributed according to the proportion of enrolled children in each district. Further distributions were subsequently carried out in each district according to gender proportion. In order to meet the required number of children of each gender; private and public schools were randomly selected using a random number generator according to their proportionate representatives [18]. The sampling procedure yielded 19 public and private primary schools, representative of the schools in the city; that is, ten boys’ (five public and five private) and nine girls’ (four public and five private) schools. At the school level, one class from each grade was randomly assigned (using the bowl method); if less than 15 children were in a selected class, another class was randomly selected.

The inclusion criteria were as follows: children aged 6–12 years, enrolled in the 1^st^–6^th^ grade, and attending public or private primary school, whose native language was Arabic, and whose parents agreed to provide informed consent in writing. Incomplete responses and children whose parents declined to consent were excluded. To compensate for incomplete and absent responses and for unanticipated problems, 2000 questionnaires were distributed.

Ethical approval to carry out the study was obtained from the Research Ethics Committee of the Faculty of Dentistry, King Abdulaziz University (number 024-13). Approval was obtained from the Ministry of Education and from schools prior to the study.

### Questionnaires and procedures

This cross-sectional study ran from September 2013 until May 2014. Variables were measured using two questionnaires: one was the Arabic version of the CFSS-DS [16] and was completed by the participating children; the other was for the children’s parents.

All parents received an extended letter explaining the objectives and procedures of the study and a consent form for child dental examination. Parents who agreed to their children’s participation signed the consent form and completed a short questionnaire. The questionnaire was in Arabic, and was divided into two parts: the first part concerned the child’s socio-demographic data; the second part contained multiple-choice questions regarding general medical history, past dental history, and the child’s previous dental experiences.

An Arabic version of the CFSS-DS, previously translated from the English version [6] and preliminarily validated clinically, [15, 16] was used to examine children’s fear via self-report. It measures dental fear using 15 questions concerning various dental treatment conditions and situations (e.g., “injections,” “opening the mouth,” “choking’). Responses described fear using a 5-point Likert scale (1 = *not afraid at all,* 5 = *very afraid*). Possible scores range from 15 to 75; higher scores indicated greater dental fear. As the dentist carries out all dental work for patients in Arabic countries, item 15 was modified to use the word “dentist” instead of “nurse.” [15].

### Reliability

Multiple school visits were made to obtain the schools’ permission, distribute the parents’ questionnaire, and then distribute the children’s questionnaire (the CFSS-DS) for completion in class (only to children whose parents had consented). Instructions for completing the CFSS-DS were provided and the questionnaire was read out loud to each class by an investigator. Other investigators helped children with reading and completing the questionnaire independently. Children were allowed to ask for help if they did not understand any item. Children were not allowed to share answers or to complete the questionnaire in groups. Children whose parents did not indicate consent were brought to an activity room with one of the investigators where they received oral health education and coloring sheets.

All participants were assigned a serial number. Systematic random sampling was used to select children from public and private schools (507 children). These children were invited to complete the CFSS-DS a second time two weeks later. Children who participated in the retest were brought to another empty class following approval from their teacher or arrangement within the school; and the same protocol used in the first administration was used. Scores from the first and second round of testing were compared to assess test-retest reliability.

### Validity

**Criterion validity** was estimated by comparing CFSS-DS scores and children’s behavior (rated using the Frankl scale [4]) during dental examination. Behavior was assessed by four trained and calibrated dentists (intraclass correlation (ICC) = 0.82). After completing the CFSS-DS, children were brought to the activity room to join their classmates. Only the child assigned for dental examination was allowed in the classroom. Children were examined while sitting on a chair with a high backrest and following the application of infection control measures. Each child was examined by one trained and calibrated dentist. Two dentists who were blinded to the child’s CFSS-DS score assessed the child’s behavior during dental examination using the Frankl Behavior Rating Scale [4].

The Frankl Behavior Rating Scale assesses children’s behavior using a 4-point scale. “A rating of 1 = “definitely negative”: refusal of treatment, forceful crying, fearfulness, or any overt evidence of extreme negativism; 2 = “negative”: reluctance to accept treatment, uncooperative, some evidence of negative attitude but not pronounced; 3 = “positive”: acceptance of treatment; cautious behavior at times; willingness to comply with the dentist, at times with reservation, but follows the dentist’s directions cooperatively; 4 = “definitely positive”: good rapport with the dentist, interest in the dental procedures, laughter and enjoyment.” [4]. Ratings were dichotomized into negative (1 and 2) and positive (3 and 4), and compared with CFSS-DS scores.

**Construct validity** was calculated on the assumption that greater dental fear as assessed by the CFSS-DS would be associated with greater fear of going to the dentist soon. A single question (“How afraid are you of going to the dentist soon?”) was added to the questionnaire in Arabic; responses used a 5-point Likert scale to match the CFSS-DS.

### Statistical analysis

All collected questionnaires were checked for completeness. Statistical analysis was carried out using the Statistical Package for Social Sciences, version 18 (SPSS Inc., Chicago, Il, USA). Statistical significance was set at *p* < 0.05. Descriptive statistics, means, and standard deviations were calculated. Each child’s total fear score was calculated by summing the scores assigned to each item of the CFSS-DS.

Internal consistency was evaluated using Cronbach’s alpha for the total sample; regarding the test-retest reliability subsample, scores from the first and second tests were compared using ICC.

Criterion validity: CFSS-DS scores of children with negative and positive dichotomized behavior ratings were compared using *t*-test.

Construct validity: Spearman’s rho was used to examine the correlation of total fear scores and scores on the single question “how afraid are you of going to the dentist soon?”

The factor structure of the Arabic version of the CFSS-DS was examined using factor analysis with principal components and varimax rotation. The Kaiser-Meyer-Olkin measure (KMO) was used to determine if the sample was appropriate for factor analysis.

In addition, *t*-test or analysis of variance (ANOVA) were used to compare age between boys and girls, and fear scores according to gender, previous dental treatments (yes or no), and type of treatment administered in the previous dental visits. Pearson’s *r* was used to assess correlation between CFSS-DS scores and age.

## Results

Of the 2000 questionnaires distributed, 1670 were returned, giving a response rate of 84 %. Fifty-three of these were excluded due to parents’ failure to sign the consent form, missing data, or children’s refusal to participate. Seventy-one children were absent on the day of examination, resulting 1546 participants (748 girls and 798 boys). Regarding the test-retest subsample, 60 children were absent for the retest, resulting in 447 participants.

The total number of participants was 1546; their mean age was 9.26 ± 1.83 years. Boys accounted for 51.62 %, with a mean age of 9.33 ± 1.82 years; the girls’ mean age was 9.19 ± 1.83 years. No significant difference was found in age between girls and boys (p = 0.125). The sample was 74.45 % Saudi, 13.91 % Yemeni, 3.23 % Palestinian, 2.47 % Syrian, 1.90 % Sudanese, 1.81 % Egyptian, 1.81 % Jordanian, and 0.45 % other Arabic nationalities such as Lebanese, Moroccan and Somali.

The mean total fear score for all participating children was 26.09 ± 10.70. A weak significant correlation was found between CFSS-DS score and age (*r* = 0.16, *p* < 0.001). There was a statistically significant higher mean total fear score in girls (29.50 ± 12.34) than in boys (22.89 ± 7.61, *p* < 0.001).

Mean CFSS-DS item scores are shown in Table 1. The most frightening items for all children and girls were “injections” followed by “dentist drilling,” “choking” then “having a stranger touch you.” In contrast, boys’ items were ranked “injections,” then “choking,” “dentist drilling,” and “having a stranger touch you.” Girls had significantly higher mean item scores than boys for all items (*p* < 0.001).

**Table 1:** CFSS-DS mean item scores and standard deviations (SD) for all children, girls, and boys

Item	Total (*N* = 1546)	Girls (*N* = 748)	Boys (*N* = 798)	*t*-test (*p* value)
	Mean (SD)	Mean (SD)	Mean (SD)	
1. Dentists	1.63 (1.02)	1.85 (1.19)	1.42 (0.77)	71.7 (<0.001)*
2. Doctors	1.47 (0.92)	1.60 (1.07)	1.34 (0.74)	30.6 (<0.001)*
3. Injections	2.49 (1.45)	2.73 (1.53)	2.27 (1.33)	39.7(<0.001)*
4. Somebody examines your mouth	1.37 (0.85)	1.55 (1.05)	1.20 (0.56)	66.1(<0.001)*
5. Having to open your mouth	1.47 (0.93)	1.67 (1.10)	1.28 (0.69)	70.2(<0.001)*
6. Having a stranger touch you	2.06 (1.35)	2.35 (1.47)	1.78 (1.16)	71.1(<0.001)*
7. Having somebody look at you	1.56 (1.05)	1.74 (1.22)	1.39 (0.81)	42.4(<0.001)*
8. The dentist drilling	2.22 (1.39)	2.58 (1.51)	1.89 (1.19)	100.0(<0.001)*
9. The sight of the dentist drilling	1.92 (1.28)	2.21 (1.43)	1.65 (1.04)	76.9(<0.001)*
10. The noise of the dentist drilling	1.76 (1.18)	2.02 (1.33)	1.53 (0.95)	69.9(<0.001)*
11. Instruments in your mouth	1.80 (1.21)	2.16 (1.39)	1.46 (0.89)	136.4(<0.001)*
12. Choking	2.21 (1.36)	2.51 (1.45)	1.93 (1.22)	71.7(<0.001)*
13. Having to go to the hospital	1.43 (0.95)	1.55 (1.10)	1.32 (0.77)	21.8(<0.001)*
14. People in white uniforms	1.27 (0.77)	1.38 (0.96)	1.15 (0.52)	34.9(<0.001)*
15. Dentist cleaning your teeth	1.42 (0.91)	1.62 (1.09)	1.24 (0.65)	68.0(<0.001)*
Total CFSS-DS score	26.09 (10.70)	29.50 (12.34)	22.89 (7.61)	163.05 (<0.001)*

*Statistically significant: *p* < 0.05

Mean item score = mean of 5 Likert points

Mean total CFSS-DS score = mean sum of scores on all 15 items

Parents’ responses indicated that 1220 participating children had previous dental experience (78.91 %) (Table 2). Mean fear scores for children with or without previous dental experience showed no significant difference (*p* = 0.46). Parents’ reports indicated the following types and percentages of treatment in previous dental visits: examination (51.28 %), restoration (46.89 %), dental extraction (41.55 %), and other less frequent treatments such as orthodontic, periodontal, and trauma management. Children’s mean fear scores showed no significant differences among the various types of previous dental treatment (*p* = 0.33).

**Table 2:** Mean fear score self-reported by children distributed according to dental history reported by parents

	N (%)	Mean (SD)	95 % CI	Min - max	Test value (*p* value)
Previous dental experience					
Yes	1220 (78.91)	26.19 (10.62)	25.60–26.79	15–75	0.538^b^ (0.46)
No	326 (21.09)	25.70 (10.99)	24.51–26.90	15–75	
Total	1546 (100)	26.09 (10.70)	25.55–26.62	15–75	
Previous dental treatments^a^					
Examination	643 (51.28)	26.14 (10.49)	25.32–26.95	15–72	1.158^c^ (0.33)
Restoration	588 (46.89)	25.53 (10.15)	24.71–26.36	15–75	
Extraction	521 (41.55)	27.20 (11.35)	26.22–28.17	15–75	
Local anesthesia	274 (21.85)	26.57 (10.14)	25.36–27.77	15–75	
Prophylaxis	209 (16.67)	26.36 (9.89)	25.02–27.71	15–74	
Endodontic	3 (0.20)	27.00 (4.58)	15.62–38.38	22 - 31	
other	9 (0.60)	28.78 (11.49)	19.95–37.61	15–50	

*SD* Standard Deviation

^a^Children may have had more than one treatment

^b^ *t*-test

^c^Analysis of variance

### Reliability

Regarding internal consistency reliability, Cronbach’s α was 0.88. Regarding test-retest reliability, the sample consisted of 50.50 % boys; the mean total CFSS-DS score at the initial test and at retest were 27.80 ± 12.99 and 26.50 ± 12.46, respectively, showing high reliability (ICC = 0.83, *p* < 0.001).

### Validity

**Construct validity** was assessed by examining the correlation between ratings of the item “fear of going to the dentist soon” with total CFSS-DS scores; a significant moderate correlation was found (*r* = 0.53, *p* < 0.001).

**Criterion validity**: The mean fear score of children with negative behavior (31.12 ± 15.69) was significantly higher than that of children with positive behavior (25.89 ± 10.41, *t* = 13.67, *p* < 0.001).

**Factor analysis** The Kaiser-Meyer-Olkin measure value was 0.93, indicating that the sample was suitable for factor analysis. The factor structure is shown in Table 3. Three factors had eigenvalues >1.00, accounting for 53.47 % of all variance. These factors were as follows: Factor 1, “fear of invasive dental procedures” (22.48 % of variance; six items, e.g., “injections” and “drilling.”) Factor 2, “fear of less invasive dental procedures” (20.39 % of variance; six items, e.g., “opening your mouth” and “somebody examining your mouth.”) Factor 3, “fear of strangers” (10.60 % of variance, three items, e.g., “dentists,” “somebody looking at you” and “a stranger touching you.”)

**Table 3:** Rotated CFSS-DS factor matrix for all children (*N* = 1546)

Item	Factor 1	Factor 2	Factor 3
1. Dentists	0.285	−0.254	**0.405**
2. Doctors	0.176	**0.591**	0.255
3. Injections	**0.616**	0.172	0.106
4. Having somebody examine your mouth	0.291	**0.616**	0.194
5. Having to open your mouth	0.359	**0.586**	0.178
6. Having a stranger touch you	0.209	0.202	**0.756**
7. Having somebody look at you	0.064	0.355	**0.758**
8. The dentist drilling	**0.781**	0.222	0.125
9. The sight of the dentist drilling	**0.728**	0.267	0.124
10. The noise of the dentist drilling	**0.657**	0.354	0.153
11. Having somebody put instruments in your mouth	**0.633**	0.369	0.147
12. Choking	**0.605**	0.202	0.216
13. Having to go to the hospital	0.257	**0.679**	0.034
14. People in white uniforms	0.178	**0.698**	0.093
15. Having the dentist clean your teeth	0.430	**0.594**	−0.056
% of accounted variance	22.48	20.39	10.6

The highest loading for each item is presented in bold

Factor 1: Fear of invasive dental procedures

Factor 2: Fear of less invasive dental procedures

Factor 3: Fear of strangers

## Discussion

The CFSS-DS is the most commonly used measure of child dental fear [5, 7]. Validating various versions of the CFSS-DS is likely to improve the understanding of dental fear by identifying and excluding culture differences [14]. The Arabic version has received preliminary validation in a clinical setting using a non-representative convenience sample; however, validation using a more representative sample, and specifically including dental avoiders who do not attend dental clinics, remained necessary [16].

This research evaluated the reliability and validity of the Arabic version of the CFSS-DS, using a representative sample of schoolchildren. High internal consistency was found (0.88), corroborating the clinically based study (0.86) [15]. The internal consistency of the Arabic version of the CFSS-DS is consistent with versions in other languages, for which values ranging from 0.83 to 0.92 have been obtained [3, 8–10, 12, 13, 19]. This indicates that the scale is a homogenous and highly reliable method of measuring Arabic-speaking children’s dental anxiety or fear. Additionally, it indicates that the Arabic questions are very specific and clearly understood by children in this age group, similar to in other languages [3, 13].

This study found high test-retest reliability for the Arabic version of the CFSS-DS (ICC = 0.82), indicating that participants’ responses are reproducible, stable, and highly correlated [20]. This finding matches the high test-retest reliability (0.86) observed in a clinical sample in the same community, [16] and it is within the range of values reported regarding other versions of the CFSS-DS (0.71–0.90) [3, 10]. The Arabic version of the CFSS-DS is therefore supported as a reliable tool for measuring dental fear in children not only in clinical settings, [16] but also in the general population.

Construct validity was tested by examining the correlation between CFSS-DS scores and “fear of going to the dentist soon”; a significant moderate correlation was found. This resembles other studies’ findings: a significant moderate correlation was found between responses to similar single questions and scores on the Arabic version of the CFSS-DS in a clinically based sample, [16] and scores on a Japanese version of the same scale in a clinically based sample and in a school sample [10]. This indicates that higher CFSS-DS scores are associated with higher fear of going or returning to the dentist soon.

The current study assessed the criterion validity of the Arabic version of CFSS-DS. Uncooperative children who exhibited negative behavior by Frankl rating had significantly higher fear scores than cooperative ones with positive behavior. This resembles other studies’ findings: children with negative behavior had significantly higher fear scores than children with positive behavior [3, 10].

Factor analysis is a way of assessing construct validity [20]. Factor analysis uses a correlation matrix between items in a given scale to determine if a subset of items is related in a way that suggests they are measuring the general concept of interest [20] (e.g. dental fear). This research used factor analysis in order to extract factors accounting for the greatest amounts of dental fear in the Arabic version of the CFSS-DS. Three factors were extracted: Factor 1, “fear of invasive dental procedures”; Factor 2, “fear of less invasive dental procedures”; and Factor 3, “fear of strangers.” This factor structure is consistent with other versions of the scale: three factors have been extracted in the Finnish, [9] Dutch, [19] Japanese, [10] Indian (Hindi) [12] and Chinese versions, [3] although the factor sequence and content vary slightly among the various versions. The three factors found in the Finnish, Dutch, and Japanese versions were as follows: “fear of highly invasive procedures” (e.g., drilling), “fear of less invasive procedures” (e.g., having somebody examine one’s mouth), and “fear of potential victimization” or “fear of general medical aspects of treatment” (e.g., choking) [9, 10, 19]. In contrast, factors such as “fear of dental treatment or of dental care personnel and procedures,” “fear of hospital personnel,” and “fear of invasive dental procedures or drilling” were found—along with differing content and rankings—in the Indian and Chinese versions. [3, 12] These differences in factor structure between the various versions may reflect differences in culture or methodology: in some studies, the child completed the questionnaire; [3, 10] in others, the parents did. [12, 19] Parents may not be able to identify their children’s dental fear as accurately as the children themselves. [21–23] In addition, environmental and social factors may affect this difference. Nonetheless, despite these differences, the highest-ranked factor typically identifies the strongest elements of dental fear. Although the CFSS-DS is multidimensional, the scale may effectively measure a “one-dimensional concept of dental fear;” [19] in the present study, this was “fear of invasive dental procedures.” This suggestion has recently been challenged: using the Rasch model to analyze its Swedish version, the CFSS-DS was supported as multidimensional, and an adjusted one-dimensional 6-item scale was created [24].

Some differences exist between the factor analysis used in the current school-based study and that used in the clinically based study conducted in the same community [15]. In the latter, the following four factors were identified: Factor 1, “fear of usual dental procedures” (eight items), Factor 2, “fear of health care personnel and injections” (three items), Factor 3, “fear of strangers,” (two items) and Factor 4, “fear of general medical aspects of treatment,” (two items) [15]. In the present study, “dentists” had low loading (0.41) and was located in Factor 3, whereas it had high loading (0.81) and was located in Factor 2 in the clinically based study. This indicates that some children in the school sample do not or rarely visit the dentist, and therefore consider dentists as strangers. In the clinical sample, the dentist was a source of fear as children connected the expected discomfort to the person providing the treatment. In contrast, “injections” had high loading (0.62) in Factor 1 in the school sample and low loading (0.46) in Factor 2 in the clinical sample. This may reflect the setting of the questionnaire’s completion: children in school might relate their fear to the word ‘injection” itself, whereas in the dental clinic setting—where the needle is usually unseen—children may have related their fear to health care providers.

In the current study, children’s mean fear score (26.09 ± 10.70) resembles data collected by previous studies worldwide [3, 10, 12, 13]. This suggests a low level of dental fear according to the cutoff score of 32, which was used in different studies to identify children with low and high fear [8, 21, 25]. The wide range of mean fear scores may relate to cultural differences between countries, such as the presence or absence of oral health services in a specific country as seen in developing countries such as India, [12] China [3] and Bosnia [13]. In these countries, children are typically taken to the dentist when invasive treatment is needed or when they are in pain, rather than for any preventive causes. Seeking dental treatment only due to pain, which is likely to lead to painful surgical treatment, increases fear in subsequent visits [26].

The mean fear score in the current study (26.09 ± 10.70) was slightly higher than in clinically based studies in the same community (23.0 ± 7.75; 23.2 ± 8.0) [16, 21]. A more generalized school/community-based sample usually exhibits higher fear levels, as it contains dental avoiders who do not attend dental clinics due to dental fear, but who are expected to attend schools [3, 10].

In the present study, girls scored significantly higher on the CFSS-DS than boys. This finding agrees with previous research: several studies have found higher dental fear in girls than in boys [1, 3, 8, 10, 21]. In our study, this difference may reflect cultural considerations, as Arabic boys are typically raised to be brave and are not expected to declare their fears, unlike girls [21]. In contrast, some researchers have found no effect of gender on dental fear [12, 27, 28].

The relation between dental fear and previous dental experience is a controversial issue. The present study found no statistically significant difference between fear scores in children with or without previous dental experience. This supports previous studies [3, 16] that found dental fear in children to be unaffected by their history of previous dental experience. Nonetheless, other researchers have found that children with previous invasive dental experience (e.g., dental fillings, extractions, pulp therapy, or local anesthetic) had greater dental fear than those without any previous dental experience [29]. In a further divergence, Nicolas et al., [27] found that children with previous dental experience had significantly lower fear than those without. Other studies have found no connection between dental fear and the number of dental visits, [28] and no relation between the type of treatment done in the first dental visit and dental fear scores in the second dental visit [3]. That result was replicated in the present study: type of previous dental treatment did not significantly affect dental fear.

In the current study, the highest-ranked items on the CFSS-DS were “injections” and “the dentist drilling.” These findings are consistent with previous research: several other studies have reported “injections” and “drilling” as the most feared items [3, 8, 10, 15, 16, 25]. In contrast, other studies [6, 9, 13] have reported “choking” and “injections” as the most feared items. Most studies have found injections to be ranked highest, with slight differences in other items’ rankings among different studies. This indicates that children in various cultures have similar concerns with dental treatment [10, 11].

This study has some limitations. It was difficult to use a self-report questionnaire with young participants, agreeing with Ten Berge et al.; [8] to overcome this difficulty, the investigators closely assisted the children in completing the questionnaire. The authors suggest that using drawings of faces in place of the 5-point Likert scale might help young children to self-report their fear. In addition, children’s behavior was only assessed during examination, which may not adequately reflect children’s cooperativeness [10]. Nonetheless, behavioral assessment during dental treatment is also unsatisfactory, as this would exclude children who do not visit the dentist due to dental fear.

Additionally, it is difficult to compare criterion and construct validity to a concrete standard. Behavior rating scales rate the child’s reaction to dental treatment and measure situational fear. They are subjective, and fear may be difficult to assess in children who have developed coping mechanisms [30]. The Frankl rating scale, used in this study, is often considered the ideal scale in dental clinics. Its popularity in pediatric dentistry research reflects its functionality, quantified scoring, and reliability. In addition, it has a high level of agreement between observers, [31] and its validity has been supported [32]. Although most studies have assessed the CFSS-DS’s criterion validity through comparison with behavior rating scales, [33] correlations between CFSS-DS scores and behavior ratings are low to moderate [14]. Nonetheless, its correlation with another self-report measure is high [34]. Future research should assess the agreement between the CFSS-DS and other psychometric measures, rather than behavioral measures, to further validate the scale.

Finally, the CFSS-DS contains some items that are minimally related to dentistry, such as “doctors,” “going to the hospital,” “somebody looking at you,” “people in white uniforms,” and “a stranger touching you.” A 6-item short version of the scale, derived from the CFSS-DS by the exclusion of unrelated items, has recently been validated [24]. The authors suggest that obtaining cut-off scores for this short scale will make it easier to use and complete than the CFSS-DS.

Sixty-one percent of children with dental fear or anxiety also showed behavior management problems [35]. In addition, fearful children are more likely to behave negatively during dental treatment than non-fearful children are [36]. Pediatric dentists and researchers may use this validated Arabic version of the CFSS-DS to measure dental fear in Arabic-speaking children. As dental fear is among the causes of behavior guidance problems among children in the dental office, the authors recommend using the CFSS-DS as a primary diagnostic tool to help identify fearful pediatric dental patients. This will help dentists to choose suitable behavior guidance techniques during dental treatment.

## Conclusion

The Arabic version of the CFSS-DS is a reliable and valid measure to assess dental fear in Arabic-speaking children. Pediatric dentists and researchers can use this validated version of the CFSS-DS to measure dental fear in Arabic-speaking children.

### Availability of data and materials

The raw data is available from the authors to any author who wishes to collaborate with us.

## Abbreviations

ANOVA: analysis of variance
CFSS-DS: the Children’s Fear Survey Schedule-Dental Subscale
ICC: intraclass correlation
KMO: Kaiser-Meyer-Olkin

## References

[1] Carrillo-Diaz M, Crego A, Armfield JM, Romero-Maroto M (2012). Treatment experience, frequency of dental visits, and children’s dental fear: a cognitive approach. Eur J Oral Sci.

[2] Ten Berge M (2008). Dental fear in children: clinical consequences. Suggested behaviour management strategies in treating children with dental fear. Eur Arch Paediatr Dent.

[3] Ma L, Wang M, Jing Q, Zhao J, Wan K, Xu Q (2015). Reliability and validity of the Chinese version of the Children’s Fear Survey Schedule-Dental Subscale. Inter J Paediatr Dent.

[4] Frankl SN, Shiere FR, Fogels H (1962). Should the parent remain with the child in the dental operatory. J Dent Child.

[5] Porritt J, Buchanan H, Hall M, Gilchrist F, Marshman Z (2013). Assessing children’s dental anxiety: a systematic review of current measures. Community Dent Oral Epidemiol.

[6] Cuthbert MI, Melamed BG (1982). A screening device: children at risk for dental fears and management problems. J Dent Child.

[7] Al-Namankany A, De Souza M, Ashley P (2012). Evidence-based dentistry: analysis of dental anxiety scales for children. Br Dent J.

[8] Ten Berge M, Veerkamp JS, Hoogstraten J, Prins PJ (2002). Childhood dental fear in the Netherlands: prevalence and normative data. Community Dent Oral Epidemiol.

[9] Alvesalo I, Murtomaa H, Milgrom P, Honkanen A, Karjalainen M, Tay K-M (1993). The dental fear survey schedule: a study with Finnish children. Inter J Paediatr Dent.

[10] Nakai Y, Hirakawa T, Milgrom P (2005). The children’s fear survey schedule–dental subscale in Japan. Community Dent Oral Epidemiol.

[11] Arapostathis KN, Coolidge T, Emmanouil D, Kotsanos N (2008). Reliability and validity of the Greek version of the Children’s Fear Survey Schedule–Dental Subscale. Inter J Paediatr Dent.

[12] Singh P, Pandey RK, Nagar A, Dutt K (2010). Reliability and factor analysis of children’s fear survey schedule-dental subscale in Indian subjects. J Indian Soc Pedod Prev Dent.

[13] Bajrić E, Kobašlija S, Jurić H (2011). Reliability and validity of Dental Subscale of the Children’s Fear Survey Schedule (CFSS-DS) in children in Bosnia and Herzegovina. Bosn J Basic Med Sci.

[14] Aartman IH, van Everdingen T, Hoogstraten J, Schuurs AH (1998). Self-report measurements of dental anxiety and fear in children: a critical assessment. J Dent Child.

[15] El-Housseiny AA, Alamoudi NM, Farsi NM, El Derwi DA (2014). Characteristics of dental fear among Arabic-speaking children: a descriptive study. BMC Oral Health.

[16] El-Housseiny AA, Farsi N, Alamoudi N, Bagher S, El Derwi D (2014). Assessment for the Children’s Fear Survey Schedule-Dental Subscale. J Clin Pediatr Dent.

[17] Dean AG, Sullivan KM, Soe MM. OpenEpi: Open Source Epidemiologic Statistics for Public Health, Version 2.3. [Last updated on 2009 May 20]. Available from: http://www.OpenEpi.com.

[18] Bouchard GA, Brasili DS, Carlson DA, Varadharajan A (2005). Random number generator: Google Patents.

[19] Ten Berge M, Hoogstraten J, Veerkamp JSJ, Prins PJM (1998). The Dental Subscale of the Childrens Fear Survey Schedule: a factor analytic study in the Netherlands. Community Dent Oral Epidemiol.

[20] Aday LA, Cornelius LJ (2006). Designing and conducting health surveys: a comprehensive guide.

[21] El-Housseiny AA, Merdad LA, Alamoudi NM, Farsi NM (2015). Effect of child and parent characteristics on child dental fear ratings: analysis of short and full versions of children’s fear survey schedule-dental subscale. Oral Health Dent Manag.

[22] Luoto A, Tolvanen M, Rantavuori K, Pohjola V, Lahti S (2010). Can parents and children evaluate each other’s dental fear?. Eur J Oral Sci.

[23] Gustafsson A, Arnrup K, Broberg AG, Bodin L, Berggren U (2010). Child dental fear as measured with the Dental Subscale of the Children’s Fear Survey Schedule: the impact of referral status and type of informant (child versus parent). Community Dent Oral Epidemiol.

[24] Lopes D, Arnrup K, Robertson A, Lundgren J (2013). Validating the dental subscale of the children’s fear survey schedule using Rasch analysis. Eur J Oral Sci.

[25] Krikken JB, van Wijk AJ, ten Cate JM, Veerkamp JS (2013). Measuring dental fear using the CFSS-DS. Do children and parents agree?. Inter J Paediatr Dent.

[26] Pinkham JR (1995). Personality development. Managing behavior of the cooperative preschool child. Dent Clin North Am.

[27] Nicolas E, Bessadet M, Collado V, Carrasco P, Rogerleroi V, Hennequin M (2010). Factors affecting dental fear in French children aged 5–12 years. Inter J Paediatr Dent.

[28] Suprabha BS, Rao A, Choudhary S, Shenoy R (2011). Child dental fear and behavior: The role of environmental factors in a hospital cohort. J Indian Soc Pedod Prev Dent.

[29] Rantavuori K, Tolvanen M, Hausen H, Lahti S, Seppä L (2009). Factors associated with different measures of dental fear among children at different ages. J Dent Child.

[30] Folayan MO, Kolawole KA (2004). A critical appraisal of the use of tools for assessing dental fear in children. African J Oral Health.

[31] Wright GZ, Kupietzky A (2014). Behavior management in dentistry for children.

[32] Winer GA (1982). A review and analysis of children’s fearful behavior in dental settings. Child Develop.

[33] Klingberg G, Broberg AG (2007). Dental fear/anxiety and dental behaviour management problems in children and adolescents: a review of prevalence and concomitant psychological factors. Int J Paediatr Dent.

[34] Howard KE, Freeman R (2007). Reliability and validity of a faces version of the Modified Child Dental Anxiety Scale. Int J Paediatr Dent.

[35] Klingberg G, Berggren U, Carlsson SG, Noren JG (1995). Child dental fear: cause-related factors and clinical effects. Eur J Oral Sci.

[36] Baier K, Milgrom P, Russell S, Mancl L, Yoshida T (2004). Children’s fear and behavior in private pediatric dentistry practices. Pediatr Dent.

